# The YABBY Family Transcription Factor AaYABBY5 Directly Targets Cytochrome P450 Monooxygenase (CYP71AV1) and Double-Bond Reductase 2 (DBR2) Involved in Artemisinin Biosynthesis in *Artemisia Annua*

**DOI:** 10.3389/fpls.2019.01084

**Published:** 2019-09-10

**Authors:** Sadaf-Ilyas Kayani, Qian Shen, Yanan Ma, Xueqing Fu, Lihui Xie, Yijun Zhong, Chen Tiantian, Qifang Pan, Ling Li, Saeed-ur Rahman, Xiaofen Sun, Kexuan Tang

**Affiliations:** Joint International Research Laboratory of Metabolic and Developmental Sciences, Key Laboratory of Urban Agriculture (South) Ministry of Agriculture, Plant Biotechnology Research Center, Fudan-SJTU-Nottingham Plant Biotechnology R&D Center, School of Agriculture and Biology, Shanghai Jiao Tong University, Shanghai, China

**Keywords:** *Artemisia annua*, artemisinin, *CYP71AV1*, *DBR2*, sesquiterpene, YABBY family transcription factor

## Abstract

Artemisinin is an effective antimalarial sesquiterpene lactone synthesized in *Artemisia annua*. Various transcription factors have been previously reported that can influence the biosynthesis of artemisinin; however, the effect of YABBY family transcription factors on artemisinin biosynthesis was unknown. In the present study, we cloned and characterized AaYABBY5: a homolog of MsYABBY5 in *Mentha spicata* which is involved in modulating the monoterpenes, as a positive regulator of artemisinin biosynthesis in *A. annua*. AaYABBY5 was found localized to the nucleus, and its expression was found to be induced by exogenous methyl jasmonic acid (MeJA) treatment. In the dual-luciferase reporter assay, it was found that AaYABBY5 significantly increased the activities of promoters of amorpha-4,11-diene synthase (*ADS*), cytochrome P450 monooxygenase (*CYP71AV1*), double-bond reductase 2 (*DBR2*), and aldehyde dehydrogenase 1 (*ALDH1*) genes. Yeast one hybrid assay showed that AaYABBY5 directly bonds to the promoters of *CYP71AV1* and *DBR2* genes. Quantitative real-time polymerase chain reaction (qPCR) of *AaYABBY5* overexpression and *AaYABBY5* antisense plants revealed a significant increase in the expression of *ADS*, *CYP71AV1*, *DBR2*, and *ALDH1* in *AaYABBY5* overexpression plants and a significant decrease in the expression of these genes in *AaYABBY5* antisense *A. annua*, respectively. Furthermore, the results of high-performance liquid chromatography (HPLC) showed that the artemisinin and its precursor dihydroartemisinic acid were significantly increased in the *AaYABBY5* overexpression plants while *AaYABBY5* downregulation resulted in a significant decrease in the concentration of artemisinin. Taken together, these results explicitly represent that AaYABBY5 is a positive regulator of artemisinin biosynthesis in *A. annua*.

## Introduction

Malaria is the most severe form of illness in human history. Although the ratio of this killer disease has declined during the last decade, it is still an intricate problem. There were an estimated 219 million cases and 435,000 related deaths reported in 2017 according to the World Health Organization (2018). *Artemisia annua* is an annual herbaceous plant of the Asteraceae family, capable of producing a wide range of terpenoids including artemisinin. Artemisinin, a sesquiterpene lactone, is currently the best therapeutic agent against malaria-causing strains of *Plasmodium falciparum* (Weathers et al., 2006). Artemisinin-based combination therapies (ACTs) as recommended by the WHO are the best choice to cure acute malaria (WHO, 2018). It has saved millions of lives in African countries. Besides the antimalarial activities, artemisinin and its derivatives are reported to have antiviral (Romero et al., 2006), anticancer (Nam et al., 2007), and antihelmintic activities (Keiser and Utzinger, 2007). Therefore, artemisinin is considered to be a potential drug for treatment of various diseases. The Chinese pharmacologist Youyou Tu is best known for her contribution to the isolation of artemisinin, and she received the 2011 Lasker Award in clinical medicine and the 2015 Nobel Prize in Physiology or Medicine.

Plants synthesize terpenes by two pathways: the mevalonate (MVA) pathway is present in the cytosol and is regulated by 3-hydroxy-3-methylglutaryl-CoA reductase (HMGR). The 2-*C*-methyl-d-erythritol 4-phosphate (MEP) pathway is found in plastids and is regulated by 1-deoxyxylulose 5-phosphate synthase (DXS) and 1-deoxyxylulose 5-phosphate reductoisomerase (DXR). Both pathways provide the precursors for terpene biosynthesis (Vranová et al., 2013) and have been well investigated. The MEP pathway mainly generates monoterpenes and diterpenes, whereas the MVA pathway produces sesquiterpenes and triterpenes (Dubey et al., 2003).

**Figure 1:**
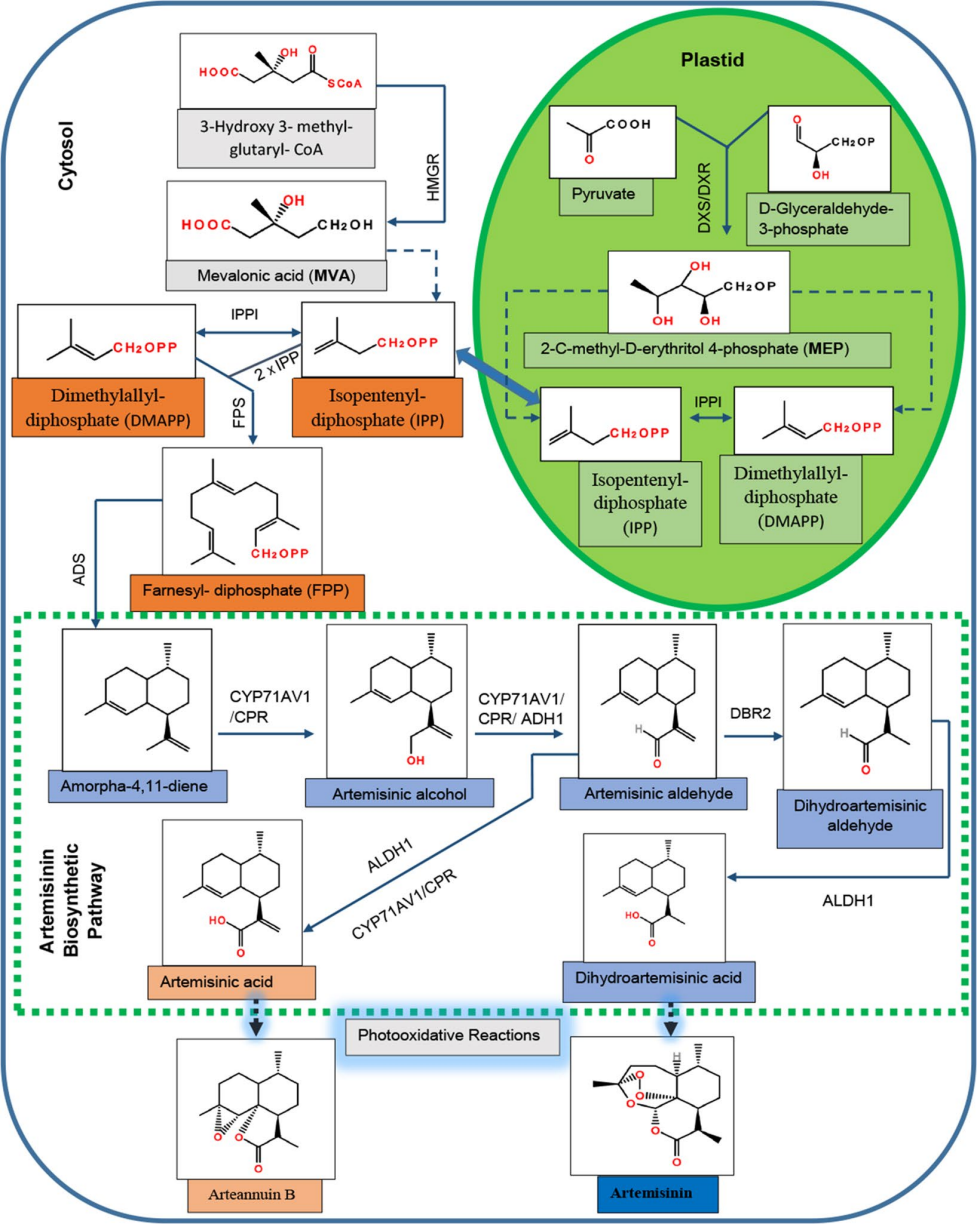
Biosynthetic pathway for artemisinin in *Artemisia annua*. HMGR, 3-hydroxy-3-methylglutaryl-CoA reductase; DXS, 1-deoxyxylulose 5-phosphate synthase; DXR, 1-deoxyxylulose 5-phosphate reductoisomerase; DMAPP, dimethylallyl diphosphate; IPP, isopentenyl diphosphate; IPPI, isopentenyl diphosphate isomerase; FPS, farnesyl diphosphate synthase; *ADS*, amorpha-4,11-diene synthase; *CYP71AV1*, cytochrome P450 monooxygenase; CPR, cytochrome P450 reductase; ADH1, alcohol dehydrogenase I; *DBR2*, double-bond reductase 2; *ALDH1*, aldehyde dehydrogenase 1.

The biosynthetic pathway of artemisinin has been almost completely elucidated (**Figure 1**). The MVA pathway produces isopentenyl diphosphate (IPP), whereas the MEP pathway generates both IPP and its isomer dimethylallyl diphosphate (DMAPP) (Towler and Weathers, 2007). IPP is isomerized to form the DMAPP in a reaction catalyzed by IPP isomerase (IPPI). In the cytosol, plant cells use one molecule of DMAPP as a primary starter molecule and two molecules of IPP as extension units to produce the farnesyl diphosphate (FPP) in a reaction catalyzed by FPP synthase (FPS) (Ma et al., 2017).

In the first committed step of artemisinin biosynthesis, FPP is converted to amorpha-4,11-diene in the presence of amorpha-4,11-diene synthase (*ADS*) (Bouwmeester et al., 1999). The successive oxidative steps following amorpha-4,11-diene yield artemisinic alcohol, and then artemisinic aldehyde is catalyzed by a multifunction cytochrome P450 monooxygenase (*CYP71AV1*) (Ro et al., 2006; Teoh et al., 2006). Artemisinic aldehyde yields two products: dihydroartemisinic aldehyde in a reaction catalyzed by double-bond reductase 2 (*DBR2*) (Zhang et al., 2008) and artemisinic acid (AA) in the presence of *CYP71AV1* and aldehyde dehydrogenase 1 (*ALDH1*). Dihydroartemisinic aldehyde yields dihydro-AA (DHAA) in the presence of *ALDH1* (Teoh et al., 2009). Finally, the photooxidative and enzyme-independent reactions using AA and DHAA as substrates generate arteannuin B and artemisinin, respectively (Brown and Sy, 2004; Brown and Sy, 2007). Although artemisinin can been obtained from AA using genetically modified yeast (Ro et al., 2006), the commercial source of artemisinin is still the plants of *A. annua*. The artemisinin content in naturally occurring *A. annua* is relatively low, approximately 0.1–1% dry leaf weight (Abdin et al., 2003); that is why its cost is high enough that most of the poorer malarial victims could not afford it (Kumar et al., 2004). Therefore, numerous attempts have been made during the last two decades to improve the yield of artemisinin in *A. annua*, using the techniques of genetic engineering. Some of them include overexpression of the genes that encode key enzymes involved in the artemisinin biosynthetic pathway (Lu et al., 2013; Shi et al., 2017) and suppression of branch pathways competing with artemisinin for the precursor molecules (Lv et al., 2016). In addition to these, transcriptional and hormonal regulations have also been studied (**Figure S1**).

Transcription factors (TFs) regulate the metabolic pathways by activating or repressing the target genes involved in the pathway (Grotewold, 2008; Iwase et al., 2009). Multiple genes are involved in the metabolic pathways, making an integrative network; therefore, it requires more time and effort to engineer each individual gene. Engineering of TFs that regulate multiple genes is a more promising and cost-effective technique (Broun and Somerville, 2001). Various TFs have been reported that targets *CYP71AV1* and *DBR2* genes involved in artemisinin biosynthesis in *A. annua*. AabHLH1 binds to the *ADS* and *CYP71AV1* promoters and activates their expression (Ji et al., 2014). AabZIP1 binds to and increases the expression of *ADS* and *CYP71AV1* genes (Zhang et al., 2015). AaMYC2 binds to the *CYP71AV1* and *DBR2* promoters and activates the gene expression (Shen et al., 2016a). The AaORA–AaTCP14 transcriptional activator complex synergistically binds to and activates the promoters of *DBR2* and *ALDH1* (Ma et al., 2018). The transcriptional regulation of artemisinin biosynthesis has also been reported to be modulated when exogenous MeJA is sprayed on plants. Examples include studies on AaMYC2, AaGSW1, and AaORA (Chen et al., 2017; Shen et al., 2016a; Ma et al., 2018). The activities of genes including *AaTCP14* have also been reported to be increased upon MeJA treatment (Ma et al., 2018). The TFs identified so far as the regulators of artemisinin biosynthesis need more knowledge to understand how they work in a regulatory network (Shen et al., 2016b).

The effect of YABBY TFs on the regulation of artemisinin biosynthesis was not previously elucidated. YABBY is a small family of TFs, specific to the seed plants (Floyd and Bowman, 2007) and encodes putative TFs defined by the presence of the C_2_C_2_ zinc finger-like domain close to the amino (N) terminal and YAB domain close to the carboxyl (C) end (Bowman and Smyth, 1999; Bowman, 2000). The YABBY domain is partially similar to the HMG domain of the high-mobility group nonhistone proteins (Bowman, 2000). Five subfamilies are recognized among angiosperms, i.e., CRABS CLAW (CRC), filamentous flower (FIL)/YABBY3 (YAB3), inner no outer (INO), YABBY2 (YAB2), and YABBY5 (YAB5) (Bowman, 2000; Yamada et al., 2004; Lee et al., 2005).

YABBYs are bifunctional TFs and can act as activators as well as repressors of secondary metabolites. Their function is dependent upon the target complex with which they interact. YABBYs have also been reported as bifunctional in the same plant (Bonaccorso et al., 2012). Previous studies report that YABBYs can regulate the secondary metabolites. Examples include the following: FIL positively regulates the anthocyanins in *Arabidopsis thaliana* (Boter et al., 2015), YABBY5 negatively regulates monoterpenes and various other terpenes in *Mentha spicata* (Wang et al., 2016), and FIL activates the genes that regulate aliphatic glucosinolate biosynthesis (Douglas et al., 2017). YABBYs play a role in diverse processes such as lamina outgrowth, cell polarity maintenance, and leaf margin establishment (Siegfried et al., 1999; Finet et al., 2016). Previous studies provided evidence that *FIL* and *YAB5* genes are involved in the formation of leaf laminar outgrowth (Siegfried et al., 1999).

Previously, it was believed that YABBY proteins bind to the DNA in a non-sequence-specific manner (Kanaya et al., 2002). Recently, several studies have proved sequence-specific binding of YABBYs (Dai et al., 2007); however, studies on YABBY binding motifs have shown that YABBY binding sites vary greatly among different species (Shamimuzzaman and Vodkin, 2013; Franco-Zorrilla et al., 2014). Protein–protein interaction studies indicate that many YABBY proteins can interact with each other; however, interactions with other TFs and corepressor complexes have also been reported (Sieber et al., 2004; Stahle et al., 2009). The regulation of YABBYs by exogenous hormones has also been noted in many plant species. In *Arabidopsis*, positive regulation of YABBYs by exogenous JA has been reported. It has been recently found that *A. thaliana* FIL/YAB1 mediates JA-induced signaling (Boter et al., 2015).

In the present study, we cloned a gene from the *A. annua* YABBY family and named it *AaYABBY5*. It was hypothesized that it might regulate the artemisinin biosynthesis by acting as either an activator or a repressor of artemisinin biosynthetic pathway genes. In a dual-luciferase (LUC) assay system, the promoters of *AaADS*, *AaCYP71AV1*, *AaDBR2*, and *AaALDH1* genes showed a significant increase in the LUC/REN activity. Furthermore, in the yeast one hybrid (Y1H) assay system, AaYABBY5 showed direct binding to the *CYP71AV1* and *DBR2* promoters. It was supposed that promoters of *AaADS* and *AaALDH1* genes might be activated by AaYABBY5 through some indirect interactions. Transgenic *A. annua* plants where *AaYABBY5* was overexpressed and downregulated, respectively, were generated, where a significant increase in the expression of *ADS*, *CYP71AV1*, *DBR2*, and *ALDH1* was found in overexpression plants, while their expression was found decreased in AaYABBY5 antisense plants. HPLC analysis further confirmed the positive role of AaYABBY5 in artemisinin biosynthesis as we obtained a significant increase in artemisinin and DHAA content in *AaYABBY5* overexpression plants and decreased artemisinin content in *AaYABBY5* antisense plants. Together, these results demonstrated that AaYABBY5 is a potent positive regulator of artemisinin biosynthesis in *A. annua*. To our knowledge, this is the first report on the regulation of artemisinin biosynthesis using AaYABBY family TFs.

## Materials and Methods

### Bioinformatic Analysis of AaYABBY5

The whole-genome database of *A. annua* developed by our lab (Shen et al., 2018) was screened using the conserved YABBY domains as input. Ten putative coding sequences were retrieved. Multiple-sequence alignment was performed using DNAMAN version 8 (Lynnon Corporation). A phylogenetic tree was constructed using protein sequences from the AaYABBY family and MsYABBY5 using the maximum likelihood method by MEGA 6.06. To evaluate the accuracy of the phylogenetic tree, bootstrap analysis was performed using 500 replicates. The evolutionary relationships among AaYABBYs, MsYABBYs, and various other plants’ YABBY members were also found.

### Cloning of *AaYABBY5*

For cloning of the *AaYABBY5* open reading frame (ORF), primers were designed for the sequences, upstream and downstream of the start and stop codons. Complementary DNA (cDNA) from leaves of *A. annua* was used as a template. The fragment was amplified using KOD plus (TOYOBO Bio-Technology, China) under the following PCR conditions: pre-denaturation at 94°C for 2 min followed by 35 cycles set at denaturation at 94°C, for 15 s, annealing at Tm-[5–10]°C for 30 s and extension at 68°C for 1 min/kb. DNA fragments were extracted using the DNA Gel Extraction Kit (AxyPrep) and ligated to the PLB vector (Tiangen Biotech, China) using the Lethal Based Fast Cloning Kit (Tiangen) using the manufacturer’s instructions. DH5α-competent cells (Invitrogen) were used for transformation. The primers used for construct preparation are listed in **Table SI**.

### Subcellular Localization of AaYABBY5

To find the subcellular localization, the ORF of *AaYABBY5* was cloned into the pEarleyGate 101 using pENTR^™^/D-TOPO^™^ cloning, according to the manual instructions. Briefly, the *AaYABBY5* ORF without termination codon was cloned in the TOPO vector (Invitrogen, Carlsbad, CA, USA) proceeded by a Gateway LR recombination reaction (Invitrogen) with pEG101. The *Agrobacterium tumefaciens* strain EHA105 harboring the construct pEG101-YABBY5-YFP was used for transient expression in leaves of 2-month-old *Nicotiana benthamiana* plants. The empty vector pEG101-YFP was used as a control. The optical density (OD) of the transformed *A. tumefaciens* culture was set at 1.5. Forty-eight hours post agroinfiltration, the yellow fluorescent signal was observed using confocal laser microscopy Leica TCS SP5 II (Leica Microsystems, Germany). The experiment was carried out in triplicate. The primers used for constructs preparation are listed in **Table SI**.

### Dual-LUC Reporter Assay

For the dual-LUC assay, effector constructs harboring the ORF of *AaYABBY5* were prepared by cloning and subsequent recombination of PCR-amplified fragments encoding AaYABBY5 into pENTR-TOPO and the Gateway destination vector pEarleyGate 104-YFP (Invitrogen, USA), respectively. To make reporter constructs for transient expression in tobacco leaves, 2,272 bp p*ADS*, 1,150 bp p*CYP71AV1*, 2,044 bp p*DBR2*, and 1,620 bp p*ALDH1* were inserted into the pGreenII 0800-LUC plasmid through *Kpn*I and *Bam*HI sites to generate *ADS*pro *: LUC*, *CYP71AV1*pro:*LUC*, *DBR2*pro:*LUC*, and *ALDH1*pro:*LUC*, according to the protocol followed by Zhang et al. (2015). The *A. tumefaciens* GV3101 strain was transformed separately with effector and reporter constructs.

Agroinfiltration and detection were performed according to the previous protocol with some modifications (Zhang et al., 2015). Briefly, a small volume of overnight agrobacterium culture harboring effector or reporter constructs was added to LB medium supplemented with appropriate antibiotics, *N*-morpholinoethane sulfonic acid (MES), and acetosyringone. After 16–24 h, the culture was centrifuged, and pellets were resuspended in magnesium chloride (MgCl_2_) to obtain an OD of 1.5. Acetosyringone was added, and the suspensions were allowed to incubate at room temperature for 3 h without shaking. The effector and reporter constructs were mixed at 1:1 concentration and infiltrated into the tobacco leaves. Negative control, i.e., the empty pEarleyGate 104-YFP mixed with the respective reporter, was infiltrated into the same leaf in the opposite direction to that of the effector strain. The experiments were carried out three times, and four independent transformations were performed for each combination of reporter and effector. Plants were kept in a dark place at room temperature for 24 h and shifted to a room with moderate light for another 24 h to allow the transgene to express. Leaf samples were collected after 48 h and analyzed by commercial dual-LUC reagents (Promega, USA) according to the manufacturer’s instructions. LUC/REN values from four independent transformations were obtained for each combination. The primers used for construct preparation are given in **Table SI**.

### Yeast One Hybrid Assay

For Y1H assay, bait constructs were prepared as follows: the full-length promoter sequences, *pADS*, *pCYP71AV1*, *pDBR2*, and *pALDH1*, were amplified and ligated into the placZ vector using ClonExpress II One-Step Cloning (Vazyme) according to instructions given in the manual. The prey constructs were prepared as follows: the ORF sequence of *AaYABBY5* was amplified and ligated into the pB42AD vector (Addgene) using a one-step cloning approach.

Experiments were performed according to the Matchmaker Gold Y1H system’s user manual (*Yeast Protocols Handbook*; Clontech, Japan). Briefly, yeast strain EGY48 was cotransformed with the bait and prey and grown on synthetic minimal double-dropout medium–tryptophan–uracil (SD-Trp-Ura). After 3 days, the colonies were picked, dissolved in sterilized 0.9% NaCl solution, and transferred to new media SD-Trp-Ura + X-Gal (5-bromo-4-chloro-3-indolyl-β-d-galactopyranoside). For each combination of bait with prey, the empty pB42AD vector cotransformed with the respective bait strain was used as a negative control. The empty bait and prey vectors were also used as the negative control. pB42AD-AaGSW1:placZ2µA was used as a positive control. The experiments were repeated three times. The primers used for construct preparation are listed in **Table SI**.

### MeJA Treatment and Relative Expression Pattern of *AaYABBY5*

To study the effect of MeJA on *AaYABBY5* expression, 6-week-old *A. annua* plants were sprayed with MeJA solution at 100-µM concentration. Control plants were treated with sterile water with 1% DMSO solution, as mock solution. Leaf samples were collected at time intervals of 0, 0.5, 1.5, 3, 6, 9, 12, and 24 h post hormonal treatment. In order to study the relative tissue-specific expression of *AaYABBY5*, 5-month-old plants of *A. annua* were used. RNA extraction and cDNA synthesis were performed using RNAprep Reagents (Tiangen) and PrimeScript RT Reagents (Takara), respectively. The expression analysis of *AaYABBY5* was performed using Roche Light Cycler 96 qPCR (Roche, Switzerland).

### Transgenic *A. annua* Plants

To generate the constructs for *AaYABBY5* overexpression in *A. annua*, the full-length coding sequence of *AaYABBY5* was amplified using PLB-*AaYABBY5* as a template. The ORF of *AaYABBY5* was cloned into the *Pst*I and *Xba*I sites of the pHB vector according to the protocol followed by Mao et al. (2005), under the control of the cauliflower mosaic virus (CaMV) *35S* promoter to generate pHB-*CaMV35Sp*-*AaYABBY5*. For downregulation of the *AaYABBY5* in *A. annua*, antisense RNA of *AaYABBY5* was used for construct preparation. For this, 412-bp *AaYABBY5* fragment upstream of the start codon and specific to only *AaYABBY5* was inserted in reverse orientation between *Bam*HI and *Xba*I sites of pHB vector. The intact pHB vector was also used for subsequent transformation into the *Agrobacterium* and then into the *A. annua* to generate the control plants (pHB only).

All the constructs were transferred to the *A. tumefaciens* strain EHA105 and introduced into *A. annua* according to the protocol (Shen et al., 2012). Briefly, sterilized seeds were placed on germination medium and cultured under the following conditions: temperature of 25°C ± 2°C, 16 h:8 h, light : dark photoperiod. Germinated seedlings were collected after 2 weeks, and 0.5-cm-diameter leaf disks were used as the explants and cocultivated with *A. tumefaciens* strain EHA105 at 25°C for 3 days. The transformed plants were selected on hygromycin containing selective media MS1, regenerated, subcultured twice, and then transferred to rooting medium MS2. The rooted plantlets were transferred to soil pots in the growth chamber. When the plants reached the age of 2 months, DNA extraction was performed, and transgenic plants were double screened based on PCR analysis using fragment-specific primers and vector-specific primers. The primer sequences are given in **Table SI**.

### RNA Extraction and Quantitative Real-Time PCR Analysis

RNA extraction was performed using RNAprep reagents (Tiangen), following the manufacturer’s instructions. Aliquots of 500-ng RNA were used for cDNA synthesis using PrimeScript RT Reagents (Takara). In order to check the expression of transcripts under study, qPCR analysis was performed following the protocol with some modifications (Shen et al., 2016a). Reagents were prepared using SYBR Green SuperReal PreMix; PCR was performed in a Roche LightCycler 96 real-time PCR machine (Roche, Switzerland) under the following conditions: initial denaturation at 95°C for 2 min, followed by denaturation at 95°C for 20 s, annealing at 54°C for 20 s, and extension at 72°C for 20 s at 40 cycles. β*-Actin* was used as an internal control for normalization of expression of all genes under study. All of the primers used in qPCR analysis are listed in **Table SI**.

### HPLC Analysis

For this analysis, 4-month-old plants of *A. annua* were used. Samples were prepared as follows: leaves were harvested from lateral branch and dried at 50°C. Leaf powder of 0.1 g was used for methanolic extraction using an ultrasonic processor adjusted at a frequency of 55 Hz at 30°C, for 30 min. The processed samples were centrifuged at 12,000 rpm for 10 min. Supernatants were collected and filtered through 0.25-μm filters. HPLC analysis was performed using a Waters Alliance 2695 HPLC system coupled with a Waters 2420 evaporative light scattering detector (ELSD) (Milford, USA). For HPLC analysis of processed samples, the previous method was followed (Lu et al., 2013).

### Statistical Analysis

Statistical significance was determined by Student’s *t*-test using paired and two-tailed distribution methods.

## Results

### Isolation and Characterization of AaYABBY5

**Figure 2:**
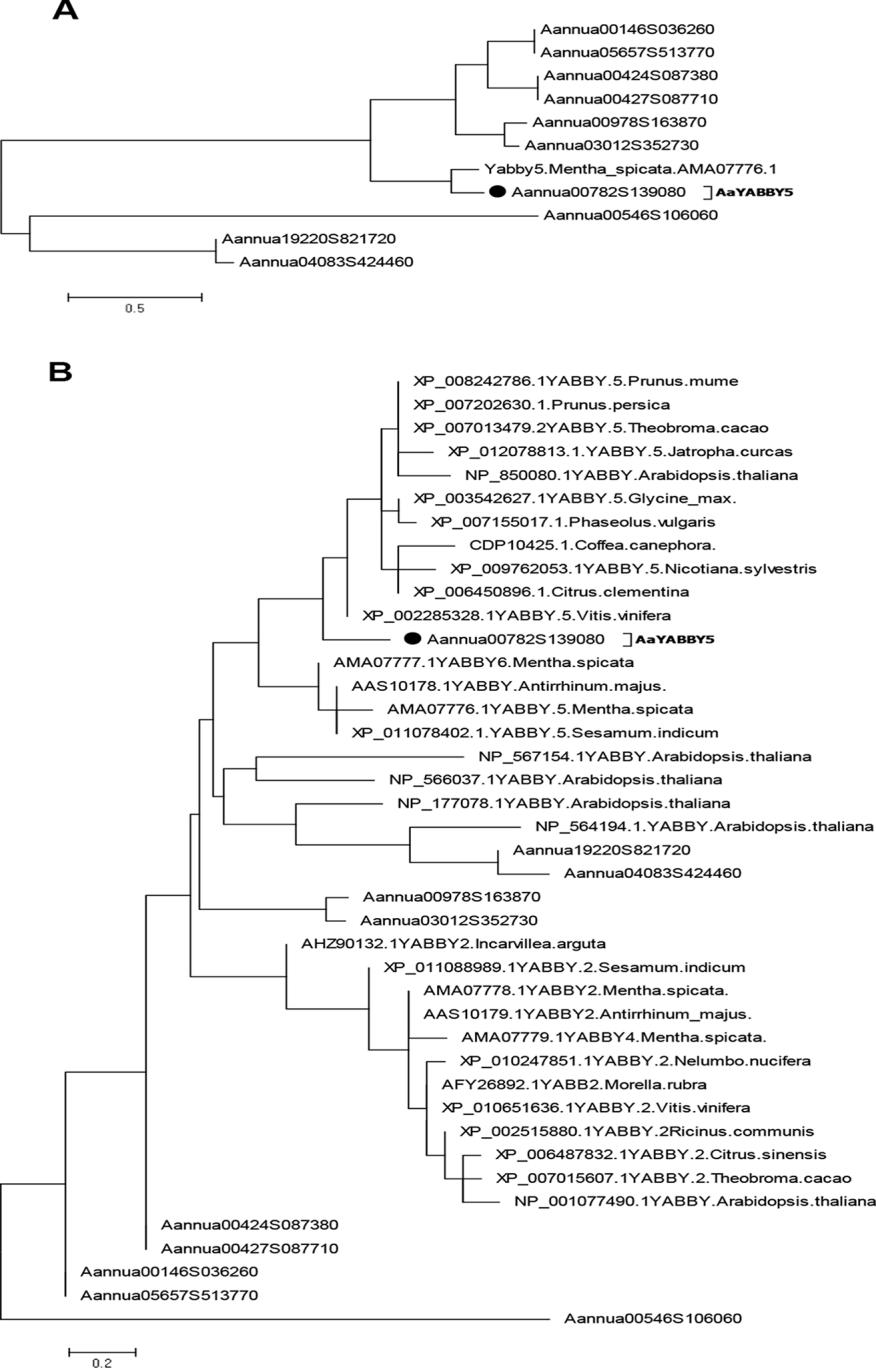
Phylogenetic analysis. The phylogenetic tree was constructed by the maximum likelihood method using MEGA 6.06. Bootstrap analysis was performed using 500 replicates. **(A)** Phylogenetic relationship was found using protein sequences from *Artemisia annua* YABBYs and MsYABBY5 protein. **(B)** Phylogenetic analysis was performed using YABBY family proteins from *A. annua*, *Mentha spicata*, *Arabidopsis thaliana*, and various other plant species. AaYABBY5 clustered among YABBY5 members of other plants. The accession number of AaYABBY5 in GenBank is MK675289.

To find the putative TFs that belong to the YABBY family, the whole-genome database of *A. annua* developed by our lab (Shen et al., 2018) was screened for YABBY family domains. We found 10 sequences that belong to the YABBY family. Multiple-sequence alignment of these sequences, using DNAMAN version 8 (Lynnon corporation), showed that all YABBY protein sequences shared a common C_2_C_2_ Zinc finger domain near the amino (N) terminal and YAB domains close to the carboxy (C) terminal like the characteristic YABBY family proteins (Bowman and Smyth, 1999; Bowman, 2000) (**Figure S2**). In order to select the TFs from the YABBY family in *A. annua*, which might be involved in the regulation of artemisinin, we searched for a homolog of MsYABBY5, a negative regulator of monoterpenes in *M. spicata* (Wang et al., 2016), based on phylogenetic analysis using protein sequences from the AaYABBY family and MsYABBY5. Only one YABBY member shared the same node with MsYABBY5 (**Figure 2A**). We then generated the phylogenetic tree using all YABBY TFs from *A. annua*, *M. spicata*, *A. thaliana*, and various other plants. AaYABBY5 clustered among the YABBY5 members and showed the closest evolutionary relationship to *Vitis vinifera* YABBY5 (**Figure 2B**). Based on the homology to *YABBY5* genes, the *AaYABBY* sequence was cloned and named as *AaYABBY5* after sequence analysis. The *AaYABBY5* gene contains an ORF of 621 bp encoding 206 amino acids. The theoretical isoelectric point and molecular weight calculated using ExPASy are 5.15 and 51.709 kD, respectively.

### Subcellular Localization of AaYABBY5 Protein

**Figure 3:**
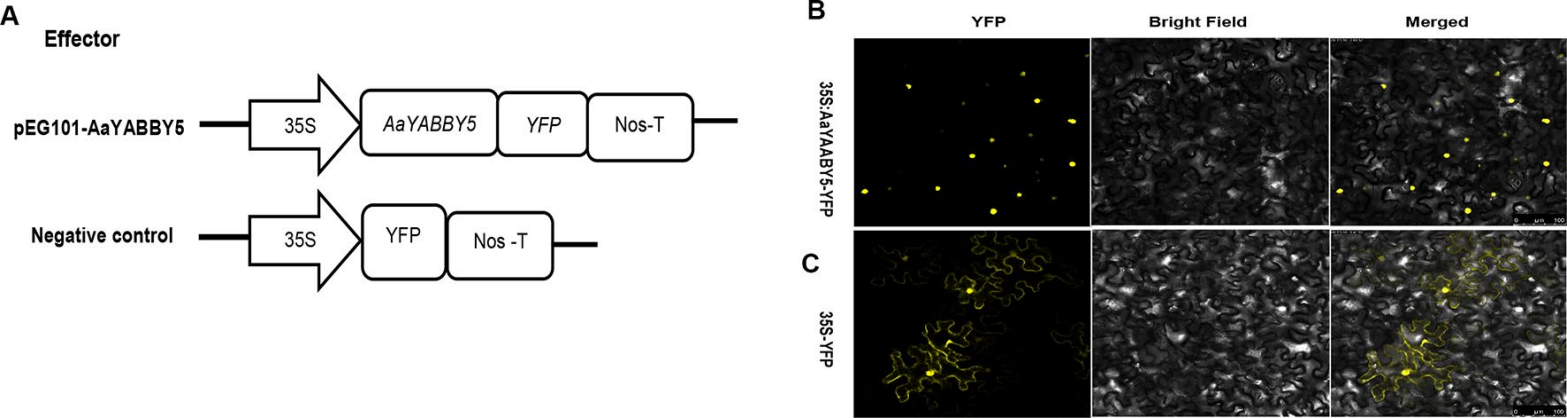
Subcellular localization of AaYABBY5 protein. **(A)** The sketch map shows the constructs used for finding subcellular localization. **(B)** Subcellular localization found under confocal laser microscope using leaf cells of *Nicotiana benthamiana* after infiltration. AaYABBY5 was found localized to the nucleus in *N. benthamiana* leaf cells infiltrated with *Agrobacterium* strains containing the coding sequence of *AaYABBY5* fused with YFP in the pEG101 vector. **(C)** Empty vector, i.e., YFP alone signal, was found in both the nucleus and cytoplasm of leaf cells agroinfiltrated with control. Bars represent 100 µm.

Sequence analysis of AaYABBY5 protein using the Swiss Institute of Bioinformatics (SIB) showed that it contains a putative nuclear localization signal (NLS). In order to determine the subcellular localization of AaYABBY5 in plant cells, an *in vivo* targeting experiment was performed in *N. benthamiana* leaf cells. The coding sequence of *AaYABBY5* was fused in-frame with the YFP, in pEarleyGate 101-YFP, where the *AaYABBY5*-YFP fusion gene was driven by the *35S* promoter. The *A. tumefaciens* strain EHA105 was used for infiltration into leaf cells of tobacco (**Figure 3A**). After 48 h post agroinfiltration, leaf samples were analyzed by confocal laser microscopy (Leica Microsystems, Germany). For AaYABBY5-YFP fusion, a strong yellow fluorescence was observed in the nucleus of *N. benthamiana* cells (**Figure 3B**), whereas a YFP-alone signal was found in both the nucleus and cytosol (**Figures 3C**). The results of the *in vivo* targeting experiment were consistent with those of sequence analysis, suggesting that AaYABBY5 is a nuclear-localized TF.

### AaYABBY5 Significantly Activated the Promoters of *ADS, CYP71AV1, DBR2*, and *ALDH1 In Vivo*

In order to test whether AaYABBY5 protein can influence the activities of promoters of the four key enzymes (ADS, CYP71AV1, DBR2, and ALDH1) involved in the artemisinin biosynthetic pathway, we searched for YABBY binding sites in them. All of the promoters of *ADS*, *CYP71AV1*, *DBR2*, and *ALDH1* contain at least one YABBY binding site given by Shamimuzzaman and Vodkin (2013) and Franco-Zorrilla et al. (2014); however, the *DBR2* promoter was found enriched with the YABBY binding motifs. Therefore, a transient dual-LUC reporter assay was performed in the *N. benthamiana* leaf system.

**Figure 4:**
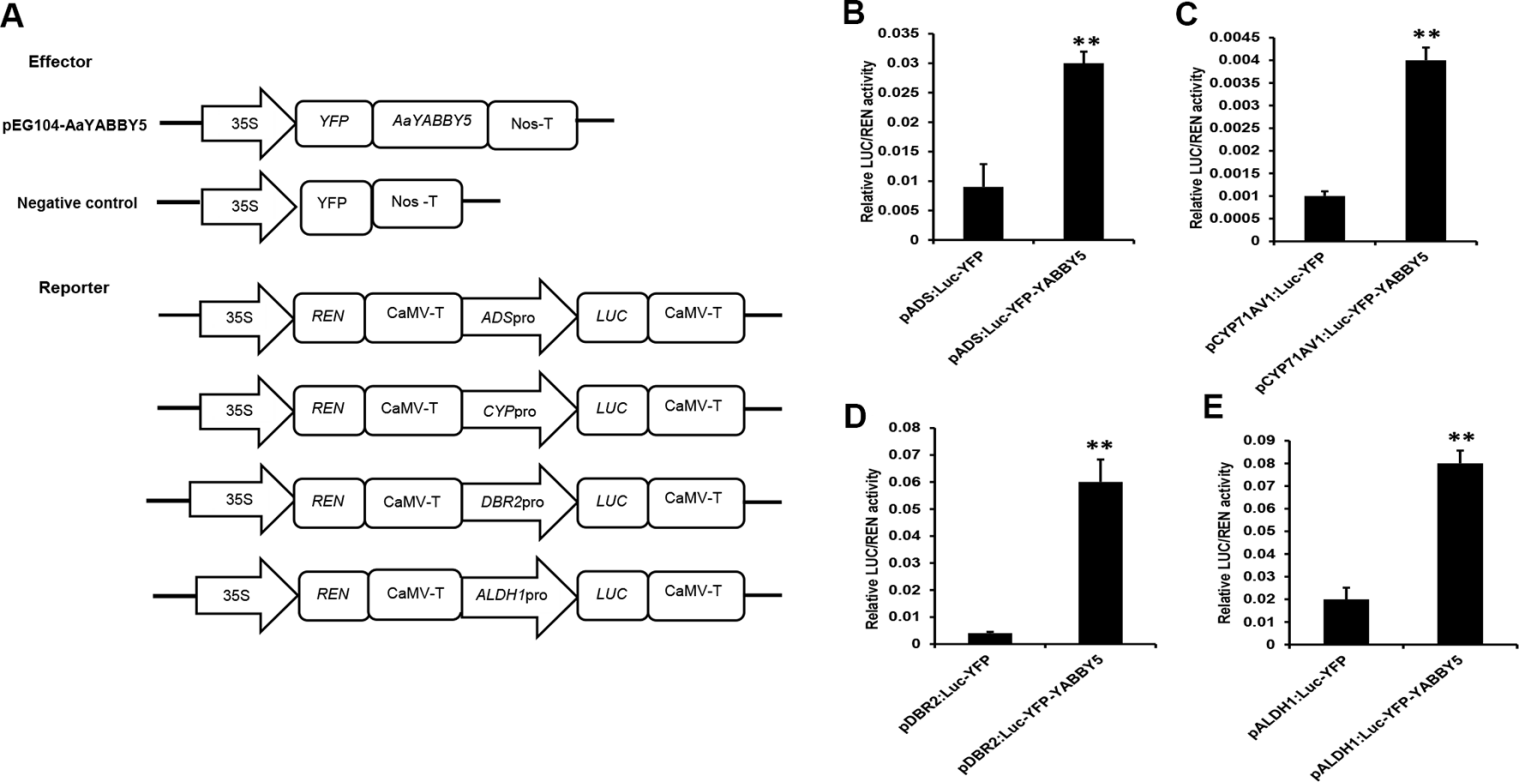
Dual-luciferase (LUC) reporter assay. **(A)** The sketch map shows the constructs for dual-LUC assay. The coding sequence of *AaYABBY5* was fused to the yellow fluorescent protein (YFP) present in the pEG104 vector to make the effector construct. Empty vectors were used as negative controls. Promoters of *ADS*, *CYP71AV1*, *DBR2*, and *ALDH1* were fused with the N-terminal of the *LUC* gene for the preparation of reporter constructs. **(B–E)** Dual-LUC analysis was performed by transient infiltration of *Nicotiana benthamiana* leaves with equal concentrations of *Agrobacterium* GV3101 cells transformed with effector and reporter constructs, respectively. The values were obtained as a ratio of the activity of firefly luciferase and renilla luciferase. A significant increase in the activities of *ADS*, *CYP71AV1*, *DBR2*, and *ALDH1* promoters was found in the presence of the AaYABBY5 effector. Data represent values obtained as mean ± SD of four independent infiltrations. Error bars represent standard deviation for *n* = 4. Statistical significance was performed using Student’s *t*-test with paired and two-tailed distribution methods. **P < 0.01.

*AaYABBY5* was cloned in pEarleyGate 104-YFP to be used as an effector. Four reporter constructs were used: pGreenII 0800-*ADS*-LUC, pGreenII 0800-*CYP71AV1-*LUC, pGreenII 0800-*DBR2-*LUC, and pGreenII 0800-*ALDH1*-LUC (**Figure 4A**). After culture preparation, agroinfiltration and detection were performed according to the previous protocol with some modifications (Zhang et al., 2015). Forty-eight hours post agroinfiltration, leaf disks were isolated from infiltrated leaves and analyzed by commercial dual-LUC reaction reagents (Promega, USA). We found a significant increase in the LUC/REN values for the four promoters under study. AaYABBY5 showed 3.9-fold increase in the activity of the *ADS* promoter, a 2.7-fold increase in *CYP71AV1* activity, and 15-fold and 3.4-fold increase in the activities of promoters of *DBR2* and *ALDH1*, respectively (**Figures 4B–E**). These values are calculated relative to the values obtained for each combination of negative control with the respective reporter construct. The LUC/REN values correspond to the LUC expression driven by the *ADS*, *CYP71AV1*, *DBR2*, and *ALDH1* promoters in the presence of AaYABBY5 protein and the presence of only empty vectors in the case of control, *in vivo*. The Renilla LUC (*REN*) gene in the pGreenII 0800-LUC vector was used as an internal control.

Although many other TFs have been reported that can activate these promoters in transient *N. benthamiana* expression system, most of them were not able to activate all the *ADS*, *CYP71AV1*, *DBR2*, and *ALDH1* promoters. They can significantly activate two of these promoters at maximum. Examples include the following. AabZIP1 can activate the *ADS* and *CYP71AV1* (Zhang et al., 2015). AaGSW1 can only activate the *CYP71AV1* (Chen et al., 2017). In the present work, the AaYABBY5 significantly activated all the four promoters under study. These results inferred that AaYABBY5 might have a strong potential to increase the artemisinin content in *A. annua*.

### AaYABBY5 Directly Binds to *CYP71AV1* and *DBR2* Promoters

Transactivation assay using the *N. benthamiana* leaf system showed that AaYABBY5 resulted in a significant increase in the activities of the promoters of *ADS*, *CYP71AV1*, *DBR2*, and *ALDH1* genes *in vivo*. To further validate the results for the molecular basis of this regulation, a Y1H assay was performed using yeast strain EGY48 co-transformed with bait (placZ-promoter sequences) and prey proteins (pB42AD-AaYABBY5). The transformants were selected on SD-Trp-Ura + X-gal media. Positive and negative controls (pB42AD:placZ promoter sequences) were used on each plate.

**Figure 5:**
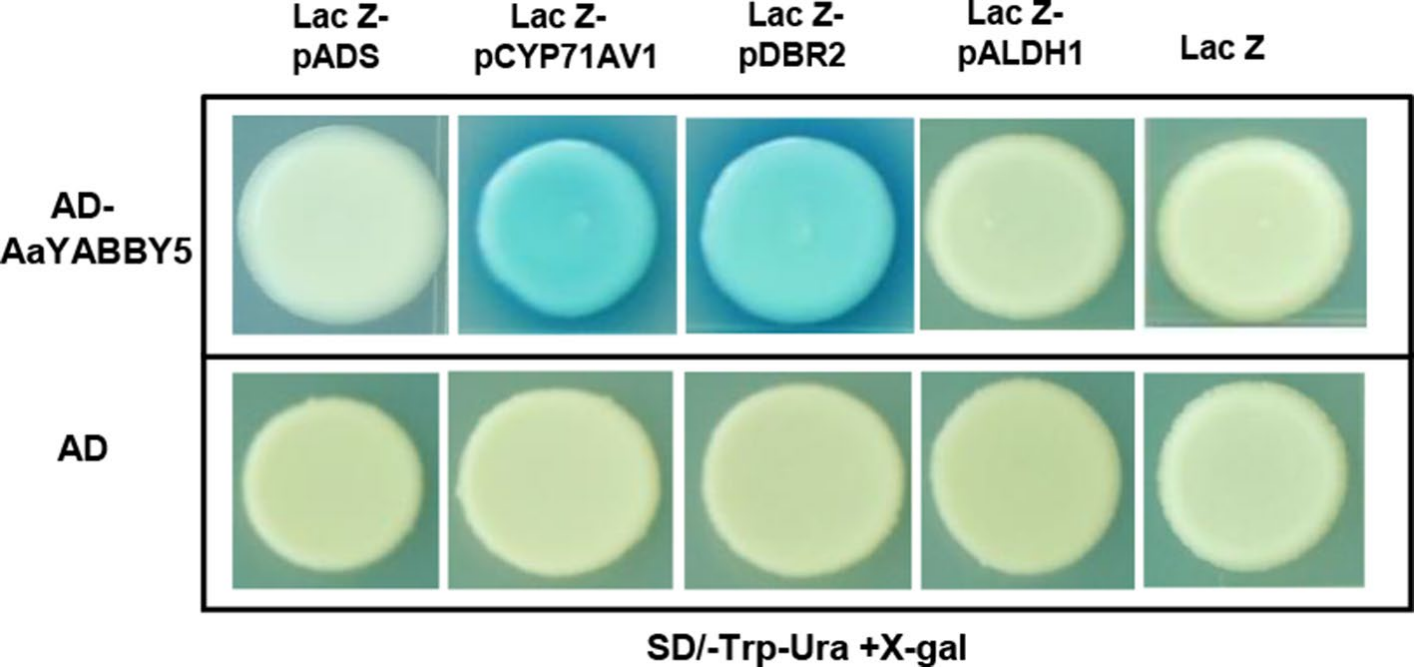
Yeast one hybrid (Y1H) assay. Y1H assay was performed using the open reading frame (ORF) sequence of *AaYABBY5* as prey and promoters of *ADS*, *CYP71AV1*, *DBR2*, and *ALDH1* as bait. The GenBank accession numbers of *pADS*, *pCYP71AV1*, *pDBR2*, and *pALDH1* are DQ448294, FJ870128, KC118523.1, and KC118525.1, respectively. AaYABBY5 directly bound to full-length *CYP71AV1* and *DBR2* promoters in yeast cells transformed with pB42AD-AaYABBY5 and plac-Z-*CYP71AV1*/plac-Z-*DBR2*, as shown by the appearance of blue colonies, whereas no binding was observed in the case of *ADS* and *ALDH1* promoters. Yeast cells transformed with empty pB42AD in combination with placZ-*ADS*/placZ-*CYP71AV1*/placZ-*DBR2*/plac-Z-*ALDH1* and empty vectors pB42AD + placZ were used as negative controls.

In the present experiment, AaYABBY5 directly bound to full-length promoters of *CYP71AV1* and *DBR2* in yeast cells and activated the transcription of the *lacZ* gene present in their downstream (**Figure 5**). *LacZ* produces β-galactosidase, which cleaves the X-gal to a blue compound, giving a blue color to the colonies. *CYP71AV1* and *DBR2* genes have also been reported to be regulated by other TFs. AaMYC2 has been reported to bind to the *CYP71AV1* and *DBR2* promoters to activate the expression of these genes (Shen et al., 2016a). The AaORA–AaTCP14 transcriptional activator complex synergistically binds to and activates the promoters of *DBR2* and *ALDH1* (Ma et al., 2018). The binding of AabHLH1 to the *CYP71AV1* promoter has also been reported (Ji et al., 2014). We found that AaYABBY5 did not show any binding to the *ADS* and *ALDH1* promoters (**Figure 5**). The experiments were repeated three times to validate the results.

### Relative Expression Analysis of *AaYABBY5* in Different Tissues

**Figure 6:**
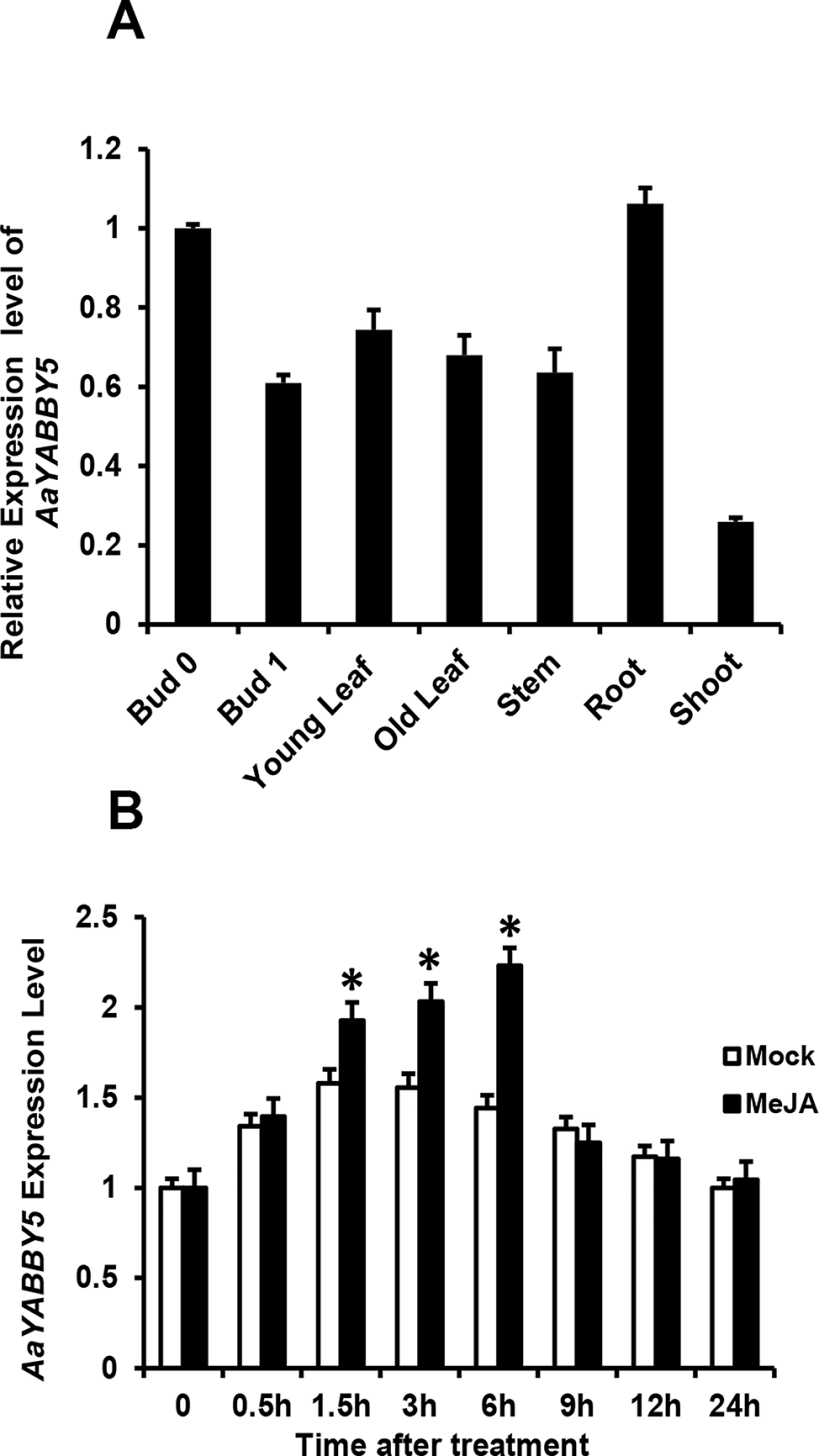
Expression and induction pattern of *AaYABBY5*. **(A)** Relative expression of *AaYABBY5* was analyzed from different tissues of 5-month-old *Artemisia annua* plants. *β-Actin* was used as an internal standard. Values represent the mean ± SD of three replicates. Error bars represent the standard deviation for *n* = 3. **(B)** *AaYABBY5* expression, in response to 100-µM MeJA, was analyzed in 4-week-old *A. annua* leaves. Water with 1% DMSO solution was used as mock solution. *β-Actin* was used as an internal standard. Values represent the mean ± SD of three experimental replicates. Error bars represent standard deviation for *n* = 3. Statistical significance was determined by Student’s *t*-test with paired and two-tailed distribution methods. Asterisks indicate the difference between treated and nontreated plants. *P < 0.05.

In order to obtain more understanding of *AaYABBY5* regulation, we checked and compared its expression in different tissues of *A. annua*, which include bud 0, bud 1, young leaf, old leaf, stem, root, and shoot (**Figure 6A**). For qPCR analysis, RNA was extracted from the tissues of 5-month-old *A. annua*, and cDNA was synthesized. We found a higher expression in bud 0 as compared to other tissues. Previously, the expression pattern of *ADS*, *CYP71AV1*, and *DBR2* genes in different tissues revealed that they show a high expression in buds and young leaves, and their expression declines progressively with the age of the leaf (Lu et al., 2013). Inconsistent with this study, we also found that *AaYABBY5* expression was higher in young leaf as compared to the old leaf stage, and it also showed maximum expression in bud 0, which declined when it progressed to bud 1 stage. However, on the contrary, *AaYABBY5* also showed a significant expression in the root, where it might have some other essential roles like defense against pathogens and worms (Köllner et al., 2008). Judd et al. (2019) reported that artemisinin is also produced by the nonglandular cells in mutant *A. annua*, which means that the regulation of artemisinin biosynthesis is not limited to only glandular trichome-specific TFs. Although AaYABBY5 is not trichome specific, it is involved in artemisinin production. These data support that AaYABBY5 potentially regulates artemisinin biosynthesis in nonglandular trichome cells.

### *AaYABBY5* Positively Responds to MeJA Treatment

Plant hormone jasmonate plays an important role in regulating plant growth and development. To check whether *AaYABBY5* responds to MeJA, the expression of *AaYABBY5* after exogenous hormone treatment was analyzed by qPCR at various time intervals (**Figure 6B**). It was found that MeJA effectively induced the expression of *AaYABBY5* in *A. annua*. The transcript level of *AaYABBY5* increased after 1.5 h of MeJA treatment, with a maximal increase obtained after 6 h, and then rapidly declined from 6 to 24 h. In model plant *A. thaliana*, it has been reported that *FIL*/*YAB3* activity can be modulated in response to exogenous jasmonate (Boter et al., 2015). Previous studies on other TFs in *A. annua* also support these results where JA signals induced the expression of *AaMYC2*, *AaGSW1*, *AaTCP14*, and *AaORA* (Shen et al., 2016a; Chen et al., 2017; Ma et al., 2018).

### *AaYABBY5* Overexpression Increases the Transcript Levels of *ADS*, *CYP71AV1*, *DBR2*, and *ALDH1* in A. *annua*, and its Downregulation Decreases Their Expression Level

**Figure 7:**
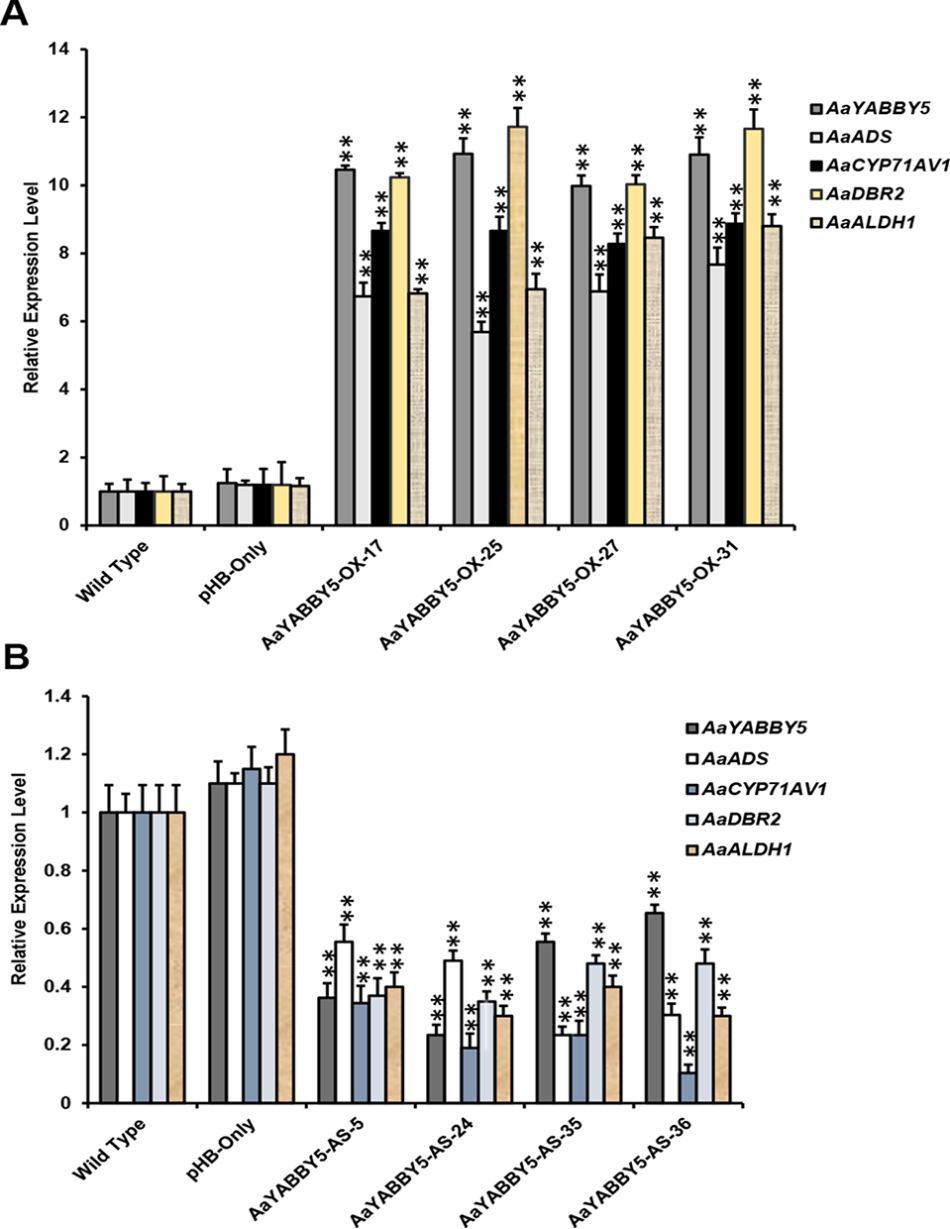
Relative expression analysis of *AaYABBY5*, *ADS*, *CYP71AV1*, *DBR2*, and *ALDH1* in *AaYABBY5* overexpression and *AaYABBY5* antisense *Artemisia annua*. **(A)** Expression of *AaYABBY5*, *ADS*, *CYP71AV1*, *DBR2*, and *ALDH1* in selected *AaYABBY5* overexpression *A. annua*. *β-Actin* was used as an internal control of each gene. Values represent mean ± SD of three experimental replicates. Error bars represent standard deviation for *n* = 3. Statistical significance was determined by Student’s *t*-test with paired and two tailed distribution methods. Asterisks indicate the difference between *AaYABBY5* overexpression and control plants. **P < 0.01. **(B)** Expression of *AaYABBY5*, *ADS*, *CYP71AV1*, *DBR2*, and *ALDH1* in selected *AaYABBY5* antisense plants of *A. annua*. *β-Actin* was used as an internal control of each gene. Values represent mean ± SD of three experimental replicates. Error bars represent standard deviation for *n* = 3. Statistical significance was determined by Student’s *t*-test with paired and two-tailed distribution methods. Asterisks indicate the difference between *AaYABBY5* antisense and control plants. **P < 0.01.

From the dual-LUC assay, we found that the AaYABBY5 protein can transactivate the promoters of *ADS*, *CYP71AV1*, *DBR2*, and *ALDH1*. The Y1H assay further provided the molecular basis of regulation of *CYP71AV1* and *DBR2* genes as we found direct binding of AaYABBY5 to their promoter regions. To study the function of AaYABBY5 in *A. annua*, we generated transgenic plants where *AaYABBY5* was overexpressed under the *35S* constitutive promoter using the pHB vector. Transgenic plants were prepared and analyzed when they reached the age of 3 months. First, we performed PCR using pHB-vector-specific forward and reverse primers, in order to confirm the vector integration. Second, we screened the plants to check *AaYABBY5* integration, using a *AaYABBY5*-specific forward primer and a pHB-vector-specific reverse primer. We obtained more than 30 transgenic plants. The *AaYABBY5* overexpression plants were analyzed for expression of *AaYABBY5* transcript levels. Four independent transgenic plants (AaYABBY5-OX-17, AaYABBY5-OX-25, AaYABBY5-OX-27, and AaYABBY5-OX-31) that showed high *AaYABBY5* expression as compared to control plants were selected for further analysis. *AaYABBY5* expression level was found 9.9-10.9 fold increased in overexpression plants as compared to the control plants. For the four artemisinin biosynthetic pathway genes, we found 5.6-7.6 fold increase for *ADS*, 8.2-8.8 fold increase for *CYP71AV1*, a remarkable 10-11 fold increase in the activity of *DBR2*, and 6.8-8.8 fold increase in the expression of *ALDH1* gene (**Figure 7A**).

To further validate the results, we generated transgenic *A. annua* plants where *AaYABBY5* gene was down-regulated using antisense RNA technology. More than 40 transgenic plants were obtained, and when they reached the age of 3 months, we performed PCR using pHB vector specific forward and reverse primers, and further screened by using antisense *AaYABBY5* RNA specific forward primer and pHB vector specific reverse primer. *AaYABBY5* transcript level was analyzed in the transgenic plants carrying antisense RNA of *AaYABBY5*. Four independent transgenic plants; AaYABBY5-AS-5, AaYABBY5-AS-24, AaYABBY5-AS-35, and AaYABBY5-AS-36 that showed decreased *AaYABBY5* expression as compared to control plants were used for further experiments. *AaYABBY5* transcript level was found 1.5-4.2 fold lower in *AaYABBY5* antisense plants as compared to the control plants. The expression level of the artemisinin biosynthetic pathway genes was found decreased as follows: *ADS*, 1.8-4.2 fold, *CYP71AV1*, 2.9-9.6 fold, *DBR2*, 2-2.8 fold, and *ALDH1*, 2.5-3.3 fold as compared to the control plants (**Figure 7B**).

### *AaYABBY5* Overexpression in *A. annua* Increases the Artemisinin Content, and It Is Decreased Under *AaYABBY5* Downregulation

**Figure 8:**
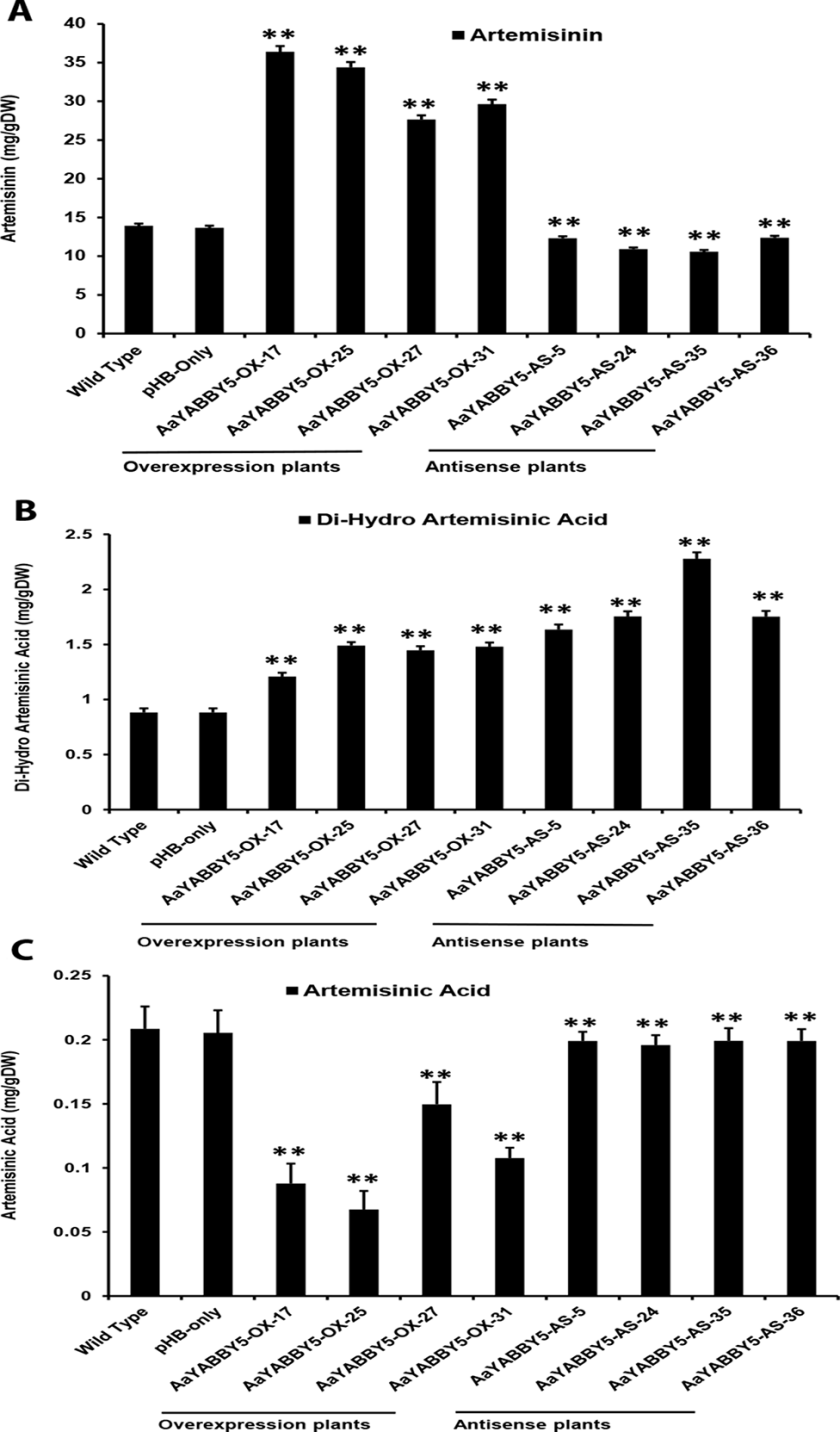
High-performance liquid chromatography (HPLC) analysis of metabolites in *AaYABBY5* overexpression and antisense plants. The concentration of **(A)** artemisinin, **(B)** dihydroartemisinic acid, and **(C)** artemisinic acid in *AaYABBY5* overexpression and antisense plants of *Artemisia annua*. Values of all metabolites represent mean ± SD of three independent experiments. Error bars represent the standard deviation for *n* = 3. Statistical significance was determined by Student’s *t*-test with paired and two-tailed distribution methods. Asterisks indicate the difference between transgenic and control plants. **P < 0.01.

The real-time PCR analysis of *ADS, CYP71AV1, DBR2*, and *ALDH1* genes revealed that their transcript levels were increased in *AaYABBY5* overexpression plants, whereas their expression was found decreased in *AaYABBY5*-antisense plants. Considering these results, HPLC analysis was performed in order to measure the concentration of artemisinin, DHAA; which is the direct precursor of artemisinin and also AA which is the precursor for arteannuin B. As expected from the study of relative expression of artemisinin biosynthetic genes, we found a significant increase in the concentration of artemisinin in the *AaYABBY5* overexpression plants, as compared to the control plants. The artemisinin concentration was found 2.66 fold, 2.52 fold, 2.02 fold, and 2.16 fold increased for AaYABBY5-OX-17, AaYABBY5-OX-25, AaYABBY5-OX-27, and AaYABBY5-OX-31 plants, respectively (**Figure 8A**). The DHAA concentration was also found significantly increased in the *AaYABBY5* overexpression plants. It was found 1.36-fold, 1.68-fold, 1.63-fold, and 1.67-fold increased for AaYABBY5-OX-17, AaYABBY5-OX-25, AaYABBY5-OX-27, and AaYABBY5-OX-31 respectively, as compared to control plants (**Figure 8B**). Conversely, AA was found decreased in the *AaYABBY5* overexpression plants and followed 2.3-fold, 3-fold, 1.3-fold, and 1.9-fold decreases in concentration as compared to the control plants (**Figure 8C**).

In the *AaYABBY5* antisense plants, artemisinin content was found decreased as compared to the control plants, and we found 1.1-fold, 1.2-fold, 1.3-fold, and 1-fold decrease in the concentration of artemisinin for the AaYABBY5-AS-5, AaYABBY5-AS-24, AaYABBY5-AS-35, AaYABBY5-AS-36, plants respectively (**Figure 8A**). Interestingly, in the selected *AaYABBY5* antisense plants, which showed decreased artemisinin content as compared to the control plants, DHAA content was found significantly higher as compared to both the control plants and *AaYABBY5* overexpression plants. The DHAA followed 1.8-fold, 1.9-fold, 2.5-fold, and 1.9-fold increases in concentration as compared to control plants (**Figure 8B**). Surprisingly, this concentration was higher as compared to that of overexpression plants and was found 1.5-fold, 1.3-fold, 1.7-fold, and 1.3-fold increased as compared to the *AaYABBY5* overexpression plants. As for AA concentration, it was found decreased in all *AaYABBY5* antisense plants, as compared to the control plants (**Figure 8C**). We found only 1 fold decrease in the concentration of AA for the four *AaYABBY5* antisense plants under study, whereas the decrease was smaller when compared to that of *AaYABBY5* overexpression plants.

## Discussion

In order to enhance the artemisinin production, various strategies have been implied so far, which can lead to increased artemisinin biosynthesis. At present, the major focus of researchers is on the transcriptional regulation of the artemisinin biosynthetic pathway, because hundreds of TFs are involved in a single signaling process. In order to add more knowledge to the transcriptional network of artemisinin biosynthesis, we selected a YABBY family TF for the present work. To our knowledge, it was not known previously whether YABBYs play a role in artemisinin biosynthesis.

We found 10 YABBY genes in the whole genome database of *A. annua* and selected a homolog of MsYABBY5, which is a regulator of monoterpenes in *M. spicata*. We cloned and characterized this gene of AaYABBY family and named it *AaYABBY5* based on its homology to YABBY5 class (**Figure 2**). It was hypothesized that the AaYABBY5 might regulate the artemisinin biosynthesis. In order to make sure that the cloned gene is a putative TF, we determined its nuclear localization (**Figure 3B**). The amino acid sequence of AaYABBY5 contains a NLS. Other TFs like AabZIP1 has also been found to be localized to the nucleus (Zhang et al., 2015).

### AaYABBY5 Directly Regulates *CYP71AV1* and *DBR2*, Whereas *ADS* and *ALDH1* Might Have Some Indirect Regulatory Mechanism Mediated by AaYABBY5

The predicted YABBY binding sites vary greatly among different species. In *A. thaliana* A/T rich repeats, whereas CC/GA rich repeats in soya bean have been defined as the possible YABBY binding motifs (Shamimuzzaman and Vodkin, 2013; Franco-Zorrilla et al., 2014). To our interest *DBR2* promoter was found enriched with both types of YABBY binding motifs; however we also found the binding motifs in other promoters, i.e., *CYP71AV1*, *ADS*, and *ALDH1*. Therefore, we performed the dual-LUC assay using AaYABBY5 effector and promoters of *ADS*, *CYP71AV1*, *DBR2*, and *ALDH1* as reporters, which inferred that AaYABBY5 could significantly increase the activities of four promoters. However, the increase was extremely high for the *DBR2* promoter. We found 3.9-fold increase in activity for *ADS*, 2.7-fold for *CYP71AV1*, 15-fold for *DBR2*, 3.4-fold for *ALDH1* promoters as compared to their respective controls (**Figures 4B–E**). This might be due to the presence of multiple YABBY binding sites on the *DBR2* promoter region, which will recruit many YABBY5 proteins to the promoter, resulting in its activation and increased expression of *LUC* gene present in its downstream.

Moreover, to find whether these promoters are directly regulated by AaYABBY5 or not, Y1H assay was performed. Here, the full-length *CYP71AV1* and *DBR2* promoters were identified as the direct targets of AaYABBY5 protein, whereas no binding was found between AaYABBY5 and *ADS* or *ALDH1* promoters (**Figure 5**). The binding of AaYABBY5 to the *CYP71AV1* and *DBR2* promoter is quite obvious as they contain YABBY binding sites. In a study performed by Chromatin Immunoprecipitation Sequencing (ChIP-Seq) and RNA Sequencing (RNA-Seq) of soya bean seedlings, it has been reported that the genes encoding cytochromes can be regulated by YABBY TFs (Shamimuzzaman and Vodkin, 2013). All that can be inferred from the present scenario is that AaYABBY5 can specifically bind to *CYP71AV1* and *DBR2* promoters to regulate them directly.

As we found in the present study, *ADS* and *ALDH1* genes are not directly regulated by AaYABBY5; however, their promoters are activated by AaYABBY5 in transiently transformed *N. benthamiana* leaf cells; therefore, we supposed that these genes might be regulated by AaYABBY5 through some indirect ways. This supposition may give rise to two possibilities: AaYABBY5 might directly bind to the promoters of those TFs which can regulate *ADS* or *ALDH1* genes, or the AaYABBY5 protein could make interactions with other proteins of *A. annua*. Some of the genes regulated by YABBYs in soya bean include *AP2* and *WRKY* (Shamimuzzaman and Vodkin, 2013). In *A. annua*, AP2 and WRKY family TFs (AaERF1/AaEFR2 and AaGSW1, respectively) have been reported as positive regulators of artemisinin biosynthesis by upregulating the expression of *ADS*/*CYP71AV1* and *CYP71AV1*/*AaORA* expression, respectively (Yu et al., 2012; Chen et al., 2017). It is possible that AaYABBY5 might regulate the *ADS* gene in *A. annua* by activating the TFs like AaERF1/AaERF2, which have proven to be the direct regulators of the *ADS* gene and can directly bind to the promoter of *ADS*. It is also possible that *ADS* and *ALDH1* genes might be indirectly regulated by AaYABBY5, through some protein–protein interactions. It has been reported that YABBY proteins form interactions with each other and also with other TFs (Sieber et al., 2004; Stahle et al., 2009).

Interestingly in a ChIP-Seq and RNA-Seq study on soya bean seedlings, *AP2* and *WRKY* genes have been identified to be regulated by YABBY family TFs (Shamimuzzaman and Vodkin, 2013). In the present study, we also found that *AaYABBY5* is positively regulated by exogenous MeJA treatment (**Figure 7B**). JA-mediated induction of YABBY genes has already been reported. In the model plant *Arabidopsis*, it has been reported recently that exogenous JA induces the activation of *FIL/YAB3*, resulting in the induction of *MYB75* and increased accumulation of anthocyanin contents, indicating that YAB1 is a positive regulator of JA signaling in *A. thaliana* (Boter et al., 2015). In *A. annua*, *AaMYC2*, *AaORA* (which belongs to the AP2/ERF family), and *AaGSW1*, which are positive regulators of artemisinin biosynthesis, have been reported to be induced by MeJA treatment (Chen et al., 2017). The similar induction pattern gives rise to the possibility of some interactions between YABBY5 protein and these TFs (**Figure S1**).

Furthermore, the regulation of artemisinin biosynthesis by the complex of TFs and their interacted protein has also been recently reported. In *A. annua*, it has been found that AaORA regulates the *DBR2* and *ALDH1* genes by making a complex with AaTCP14. AaTCP14 can directly bind to the respective promoter regions, whereas AaORA alone cannot be bound (Ma et al., 2018). AaORA belongs to the AP2 family of the TFs, and it has been reported previously that AP2 family proteins can be regulated by YABBY TFs (Shamimuzzaman and Vodkin, 2013). On the basis of the literature, it can be assumed that AaYABBY5 might regulate *ALDH1* gene by making interactions with the AaORA protein (**Figure S1**), form a complex with AaTCP14, and recruit the triplex complex to the *ALDH1* promoter region, or by some other molecular mechanism; however, these hypotheses will be further studied in future work.

Moreover, the relative expression pattern of *AaYABBY5* in different tissues (**Figure 6A**) revealed its higher expression in bud 0 as compared to bud 1, young leaf, old leaf, stem, and shoot. Similarly, *AaYABBY5* expression was found decreased in old leaf as compared to the young leaf stage. It has been reported previously that *ADS*, *CYP71AV1*, and *DBR2* genes are highly expressed in the bud and young leaves, and their expression declines progressively with the age of the leaf (Lu et al., 2013). A higher expression pattern of *AaYABBY5* in bud 0 and young leaf as compared to bud 1 and old leaf is parallel to the expression pattern followed by three key genes: *ADS*, *CYP71AV1*, and *DBR2* (Lu et al., 2013). Therefore, the regulation of *ADS*, *CYP71AV1*, and *DBR2* genes by AaYABBY5 is quite meaningful. However, the expression of *AaYABBY5* in roots was also found significant. Although most of the artemisinin-regulating genes show lower expression in roots, the higher expression of *AaYABBY5* in roots might be related to some regulatory processes taking place in the root cells, for instance, control of pathogens and worms (Köllner et al., 2008).

### Functional Analysis of *AaYABBY5* Overexpression and Antisense *A. annua* Plants Revealed Its Positive Role Towards Artemisinin Biosynthesis

The study on the selected *A. annua* plants where *AaYABBY5* was upregulated (AaYABBY5-OE-17, AaYABBY5-OE-25, AaYABBY5-OE-27, and AaYABBY5-OE-31) and downregulated (AaYABBY5-AS-5, AaYABBY5-AS-24, AaYABBY5-AS-35, and AaYABBY5-AS-36) confirmed that AaYABBY5 could potentially regulate the artemisinin-related genes. Relative expression analysis of four key genes *ADS*, *CYP71AV1*, *DBR2*, and *ALDH1* in *AaYABBY5* overexpression plants, as compared to the control plants, revealed that there was a significant increase in the expression of these genes in four *AaYABBY5* overexpression plants (**Figure 7A**). We also found a significant decrease in the expression of *ADS*, *CYP71AV1*, *DBR2*, and *ALDH1* genes in selected *AaYABBY5* antisense plants (**Figure 7B**). The significant change in the expression of these key genes in transgenic plants was found consistent with the previous studies on artemisinin regulation using other proteins/TFs. It has been found recently that the expression of *ADS*, *CYP71AV1*, *DBR2*, and *ALDH1* genes is increased in *AaTCP14* overexpression *A. annua* plants (Ma et al., 2018).

Moreover, *AaORA* overexpression in *A. annua* has also been previously reported to induce the expression of *ADS*, *CYP71AV1*, *DBR2*, *AaERF1* (Lu et al., 2013), and *ALDH1* (Ma et al., 2018). The effect of overexpression of YABBYs on the other genes involved in biosynthetic pathways has also been reported in other plants. In *A. thaliana*, it has been reported that overexpression of *FIL* increased the expression of *MYB75* (Boter et al., 2015). In *MsYABBY5*-overexpressing transgenic plants of *M. spicata*, no significant change in the expression of genes involved in monoterpene synthesis or the MEP pathway was found; moreover, in *MsYABBY5* RNAi transgenic plants, the level of *MsWRKY79* was found decreased; however, in overexpression plants, no significant increase has been reported (Wang et al., 2016). By considering the influence of AaYABBY5 on artemisinin biosynthetic genes, it was supposed to be a positive regulator of artemisinin biosynthesis.

Consistent with the relative expression analysis of *ADS*, *CYP71AV1*, *DBR2*, and *ALDH1* genes in transgenic *A. annua*, the comparative HPLC analysis of *AaYABBY5* overexpression and antisense plants as compared to the control plants revealed that in *AaYABBY5* overexpression plants, artemisinin biosynthesis was enhanced and resulted up to a maximum of 36-mg/g dry leaf weight (**Figure 8A**). This concentration is quite high and is 2.7-fold higher than that of control plants. We have also found a significant reduction in artemisinin content of *AaYABBY5* antisense plants, where it was found decreased up to 1.3-fold as compared to the control plants (**Figure 8A**). As discussed above, the expression of *ADS*, *CYP71AV1*, *DBR2*, and *ALDH1* genes was significantly increased in *AaYABBY5* overexpression plants. Moreover, we also found from a Y1H assay that AaYABBY5 can directly bind to the *CYP71AV1* and *DBR2* promoters. In the artemisinin biosynthetic pathway (**Figure 1**), CYP71AV1 is involved in two steps: first, the conversion of amorpha-4,11-diene to artemisinic alcohol and then the conversion of artemisinic alcohol to artemisinic aldehyde. We can suppose that the ability of AaYABBY5 to bind to and activate the *CYP71AV1* promoter and increased expression of this TF in overexpression plants might have speeded up the reactions that involved CYP71AV1, thus increasing the metabolic flux toward artemisinin biosynthesis.

It should be noted that an increase in the activity of *DBR2* was found dramatically high enough that it was not comparable to the activities of other promoters: *ADS*, *CYP71AV1*, and *ALDH1*. Our study also proved that the *DBR2* promoter could significantly be activated by AaYABBY5, which directly binds to this promoter sequence. Artemisinic aldehyde is the substrate which can be used by two different enzymes, thereby giving two paths; one path generates dihydroartemisinic aldehyde in the presence of the DBR2 enzyme, whereas the other one produces the AA (**Figure 1**). We can suppose that when *AaYABBY5* was overexpressed, more AaYABBY5 protein molecules were available to bind to and activate the *DBR2* promoter region. The increased activity of the DBR2 enzyme would have accelerated the reaction and directed more flux toward dihydroartemisinic aldehyde to produce more molecules of DHAA, as we found a significant increase in the concentration of DHAA in the *AaYABBY5* overexpression plants (**Figure 7B**). As a result of this, the side branch which produces AA might have repressed, as we found in **Figure 7C**; the AA was found decreased in *AaYABBY5* overexpression plants as compared to the control plants. Although the regulatory mechanism of secondary metabolites by YABBYs has not been completely elucidated, the regulation of various metabolites by YABBYs has been reported in other plants. In *A. thaliana*, FIL activates *MYB28*, which in turn activates genes involved in aliphatic glucosinolate biosynthesis. FIL also represses many TFs which regulate inflorescence architecture (Douglas et al., 2017). It has been reported that in *Arabidopsis* YAB3 results in increased accumulation of anthocyanin contents (Boter et al., 2015). In *M. spicata* it has been reported that overexpression of *MsYABBY5* lowered the monoterpenes and sesquiterpenes, whereas the reverse was obtained as a result of silencing of *MsYABBY5* using RNA interference (RNAi), showing that MsYABBY5 is a negative regulator of monoterpenes in spearmint (Wang et al., 2016). OsYABBY4 negatively regulates the expression of the gibberellin (GA) 20-oxidase 2 gene (*GA20ox2*) and SLR1 (the sole DELLA protein negatively controlling GA responses in rice).*GA20ox2* is the direct target of SLR1 in GA signaling pathway (Yang et al., 2016). By downregulating the expression of *GA20ox2* and SLR1, OsYABBY4 acts as a negative regulator of GA signaling.

The decreasing concentration of artemisinin in *AaYABBY5* antisense plants can be referred to as the decrease in the activities of enzymes CYP71AV1 and DBR2. When the expression of *AaYABBY5* was downregulated, the expressions of *CYP71AV1* and *DBR2* were also decreased; therefore, the reactions that use these enzymes might have slowed down, resulting in decreased artemisinin production as compared to the control plants. Moreover, it was found that the concentration of AA was decreased in *AaYABBY5* antisense plants (**Figure 7C**); however, this decrease was quite low as compared to that of *AaYABBY5* overexpression plants. The possible reason behind this might be that in *AaYABBY5* overexpression plants, due to the increased activity of DBR2, the flux toward dihydroartemisinic aldehyde was highly increased, which repressed the production of AA which uses the same precursor molecule. Considering these results, we can suppose that in the *AaYABBY5* antisense plants, there was no biasness, in the competing reactions, generated due to the remarkable increase in the DBR2 activity in the presence of AaYABBY5, which allowed more AA molecules to be synthesized as compared to those in *AaYABBY5* overexpression plants. The concentration of DHAA was found to be increased in the *AaYABBY5* antisense plants as compared to control and *AaYABBY5* overexpression plants (**Figure 7B**); however, the concentration of DHAA was only about 1/10 of artemisinin. As the artemisinin content was found significantly decreased in *AaYABBY5* antisense plants, this gives rise to the additional role of AaYABBY5, where it might have some function in repressing the oxidative steps leading to the formation of artemisinin from DHAA. However, the molecular basis of this function cannot be predicted because it has been known that this reaction is enzyme independent; hence, the mechanism remains unambiguous. In short, the significant improvement in the artemisinin content in *AaYABBY5* overexpressing *A. annua* proved that AaYABBY5 is a potent positive regulator of artemisinin biosynthesis.

This work is of great interest as AaYABBY5, the TF under study, was found to induce the expression of all the four genes regulating key enzymes involved in artemisinin biosynthesis and resulted in significant improvement in the concentration of artemisinin. Most of the previous works on transcriptional regulation of artemisinin biosynthesis have reported only one or two promoters as targets; however, in this case, a significant increase in the activity of four promoters and a remarkable increase in artemisinin level were found. The present study increased the knowledge about artemisinin regulatory network, as it introduced a new TF directly regulating two enzymes, CYP71AV1 and DBR2, involved in artemisinin biosynthesis and laid a foundation for further research where one can find how AaYABBY5 regulates the *ADS* and *ALDH1* genes. Furthermore, the regulation of precursor molecules, acting upstream of the artemisinin biosynthetic pathway, by AaYABBY5, can also be explored.

## Data Availability

The datasets generated for this study are available on request to the corresponding author.

## Author Contributions

S-IK and QS planned and designed the research. S-IK and XF performed the experiments. S-IK, QS, LX, YZ, CT, QP, and LL analyzed the data. S-IK wrote the manuscript. QS, YM, XF, SR, XS, and KT revised the manuscript. All authors approved the manuscript for submission.

## Funding

This work was supported by grants from the Bill & Melinda Gates Foundation (OPP1199872), the China National Key Research and Development Program (2017ZX09101002-003-002), and the Young Scientists Fund of the National Natural Science Foundation of China (Grant No. 31600231).

## Conflict of Interest Statement

The authors declare that the research was conducted in the absence of any commercial or financial relationships that could be construed as a potential conflict of interest.

## Text Footnotes

The *AaYABBY5* nucleotide sequence cloned for this study has been submitted to NCBI GenBank under accession number MK675289. The GenBank accession numbers of promoters of *ADS*, *CYP71AV1*, *DBR2*, and *ALDH1* used in the present research are DQ448294, FJ870128, KC118523.1, and KC118525.1, respectively.
