# Supplementary material for: The YABBY Family Transcription Factor AaYABBY5 Directly Targets Cytochrome P450 Monooxygenase (CYP71AV1) and Double-Bond Reductase 2 (DBR2) Involved in Artemisinin Biosynthesis in Artemisia Annua
Source: Front Plant Sci. 2019 Sep 10;10:1084. doi: 10.3389/fpls.2019.01084 (PMC6746943; doi:10.3389/fpls.2019.01084)
Supplement: Supplementary file 1 [file DataSheet_1.pdf]

Supplementary Material

Fig. S1.tif

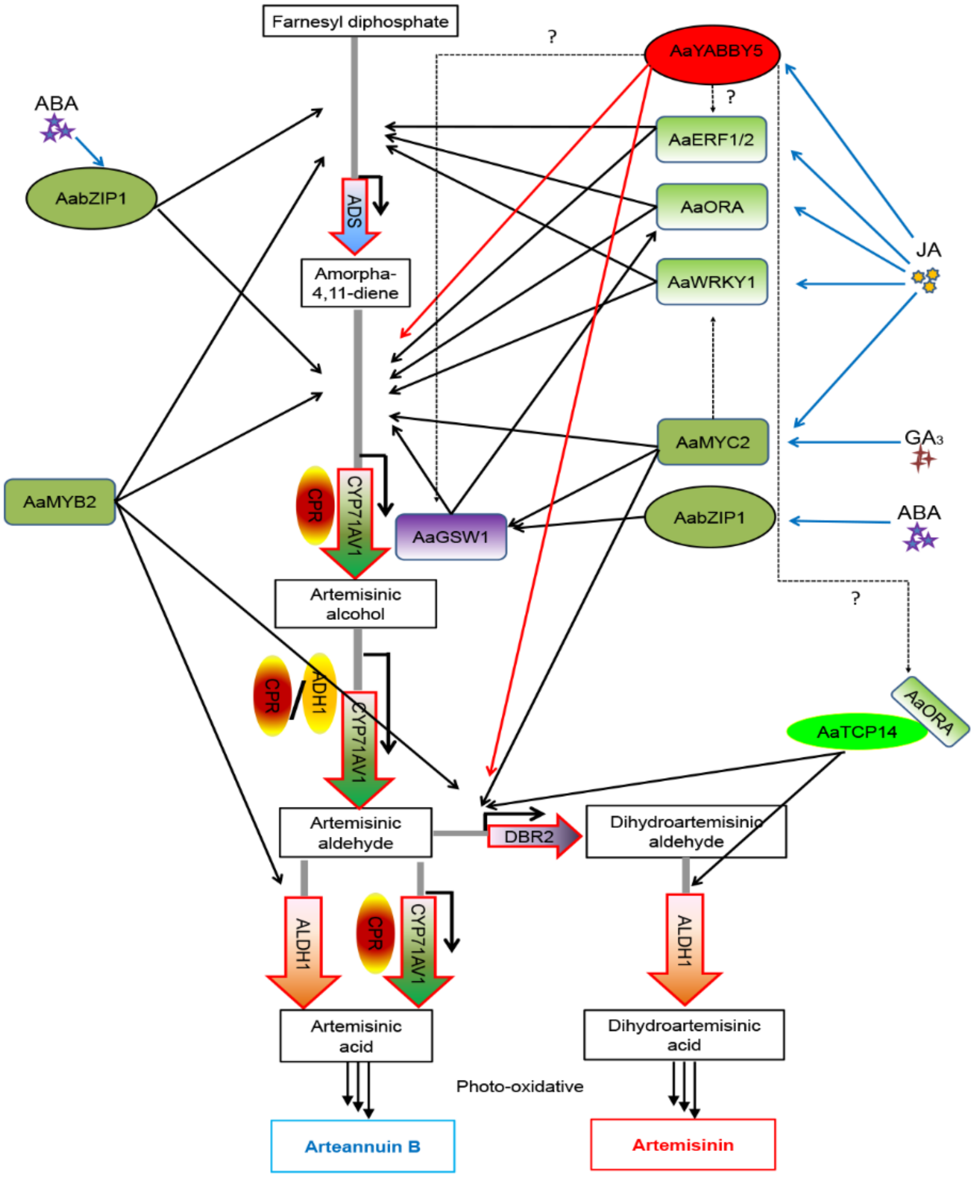

Fig. S1. Transcriptional and hormonal regulation of artemisinin biosynthetic pathway in *Artemisia annua.* Transcription factors are regulated by different hormones such as JA, ABA, GA3. These hormones induce the expression of transcription factors which then bind to the

promoters of artemisinin biosynthetic genes: *ADS, CYP71AV1, DBR2* and *ALDH1* and increase artemisinin production. JA; Jasmonic acid, ABA; Abscisic acid, GA3; Gibberelins, ADS; Amorpha-4,11-diene synthase, CYP71AV1; Cytochrome P450 monooxygenase, CPR; Cytochrome P450 reductase, ADH1; Alcohol dehydrogenase I, DBR2; Double bond reductase 2, ALDH1; Aldehyde dehydrogenase 1. Blue arrows represent regulation of transcription factors by hormones. Black arrows represent transcription factors that bind to the promoter of the genes. Red arrows represent binding of AaYABBY5 to its target genes. Black small arrows represent activation of target genes by binding of transcription factors. Dotted lines represent possible interactions between transcription factors. Question mark represents that these interactions are proposed on the basis of literature and need to be confirmed (modified from Shen et al., 2016b).

Fig.S2.tif

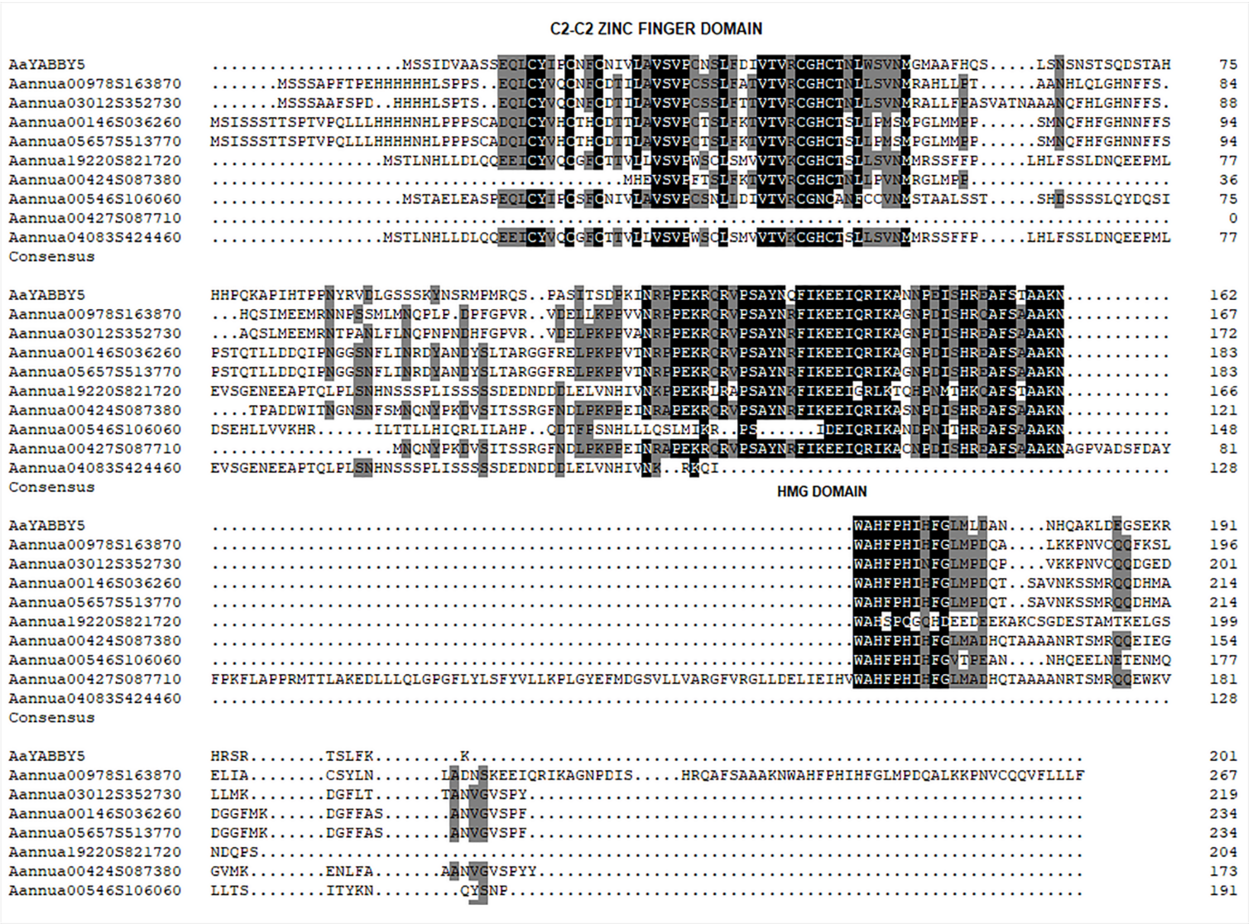

Fig. S2. Alignment of AaYABBY family protein sequences. Multiple sequence alignment of AaYABBY5 protein sequence and other YABBY proteins identified in *A. annua* performed by DNAMAN. The amino acid residues shaded in black represents 75% homology, and the grey color represents 50% homology. N terminal C2-C2 zinc finger domain and C-terminal HMG box are labelled. The accession number of AaYABBY5 in GenBank is MK675289.

Table SI. Primers Used in the Present Study

Primer	Sequence (5'→3')	Purpose
**AaYABBY5F**	5' TAAAGAGAGACTAACTTGCTTGG 3'	Cloning
**AaYABBY5R**	5' ATAAGAGGATTTGCTTTAGATTTC 3'	Cloning
**TOPO-YABBY5F**	5' CACCATGTCAAGCATAGATGTAGCAG 3'	Subcellular Localization
**TOPO-YABBY5R**	5' TTTCTTGAATAATGAGGTCCTCG 3'	Subcellular Localization
**pGreen0800-ADS-F**	5'CGGTATCGATAAGCTTCTCGAGTATGGTGTTTCAAC3'	Dual LUC Assay
**pGreen0800-ADS-R**	5'ATCCCCCGGGCTGCAGGATTTTCAAAACTTTGAATA3'	Dual LUC Assay
**pGreen0800-CYP-F**	5'CGGTATCGATAAGCTTAATGGGTCAATTTCGGGTTG3'	Dual LUC Assay
**pGreen0800-CYP-R**	5'ATCCCCCGGGCTGCAGCATGCTTTTAGTATACTC3'	Dual LUC Assay
**pGreen0800-DBR2-F**	5'CGGTATCGATAAGCTTAAGAACTTCGAGATAGAAAA 3'	Dual LUC Assay
**pGreen0800-DBR2-R**	5'ATCCCCCGGGCTGCAGTCAGTGATGGAGTTGGTAAA3'	Dual LUC Assay
**pGreen0800-ALDH1-F**	5'CGGTATCGATAAGCTTATGAACCATTAGAAGGGAAG3'	Dual LUC Assay
**pGreen0800-ALDH1-R**	5'ATCCCCCGGGCTGCAGCTTTGTTTTTATGAAATTT3'	Dual LUC Assay
**pB42AD-YAB5F**	5'GATTATGCCTCTCCCGAATTCATGTCAAGCATAGATGTAGCA 3'	Y1H Assay
**pB42AD-YAB5R**	5'GAAGTCCAAAGCTTCTCGAGTCATTTCTTGAATAATGAGGTC 3'	Y1H Assay
**ADSpro:LacZ-F**	5'TATTGGATCGGAATTCCTCGAGTATGGTGTTTCAAC3'	Y1H Assay
**ADSpro:LacZ-R**	5'GAGCACATGCCTCGAGGATTTTCAAAACTTTGAATA3'	Y1H Assay
**CYP71AV1pro:LacZ-F**	5'TATTGGATCGGAATTCAATGGGTCAATTTCGGGTTG3'	Y1H Assay
**CYP71AV1pro:LacZ-R**	5'GAGCACATGCCTCGAGCATGCTTTTAGTATACTC3'	Y1H Assay

**DBR2pro:LacZ-F**	5'TATTGGATCGGAATTCAAGAACTTCGAGATAGAAAA3'	Y1H Assay
**DBR2pro:LacZ-R**	5'GAGCACATGCCTCGAGTCAGTGATGGAGTTGGTAAA3'	Y1H Assay
**ALDH1pro:LacZ-F**	5'TATTGGATCGGAATTCATGAACCATTAGAAGGGAAG 3'	Y1H Assay
**ALDH1pro:LacZ-R**	5'GAGCACATGCCTCGAGCTTTGTTTTTATGAAATTT3'	Y1H Assay
**GK_pLacZi6778F**	5' TTCTGTTCGGAGATTACCGAATC 3'	Sequencing of constructs in placZ
**GK_pLacZi122R**	5' AAGGACCTAATGTATAAGGAAAG 3'	Sequencing of constructs in placZ
**PstI YABBY5F**	5' GCCTGCAGATGTCAAGCATAGATGTAGC 3'	*AaYABBY5* OX Plants
**XbaI YABBY5R**	5' GCTCTAGATCATTTCTTGAATAATGAGG 3'	*AaYABBY5* OX Plants
**pHB-check F**	5' GTTACCCAACTTAATCGCCTTGC 3'	Sequencing of Constructs in pHB
**pHB-check R**	5' GCGGGACTCTAATCATAAAAACC 3'	Sequencing of Constructs in pHB
**OS-pHB-AntiY5F**	5'CGAACGAAAGCTCTAGAGAGAGACTACTTGCTTGGA3'	*AaYABBY5* AS Plants
**OS-pHB-AntiY5R**	5'CTCTCTAAGCTTGGATCCGCTGTCTTTTCTCCGGGGGGCGA3'	*AaYABBY5* AS Plants
**Rt-Actin-F**	5' CCAGGCTGTTCAGTCTCTGTAT3'	qRT-PCR
**Rt-Actin-R**	5' CGCTCGGTAAGGATCTTCATCA3'	qRT-PCR
**Rt-ADS-F**	5' AATGGGCAAATGAGGGACAC 3'	qRT-PCR
**Rt-ADS-R**	5' TTTCAAGGCTCGATGAACTATG 3'	qRT-PCR
**Rt-CYP71AV1-F**	5' CGAGACTTTAACTGGTGAGATTGT 3'	qRT-PCR
**Rt-CYP71AV1-R**	5' CGAAGCGACTGAAATGACTTTACT 3'	qRT-PCR
**Rt-DBR2-F**	5' GCGGTGGTTACACTAGAGAACTT 3'	qRT-PCR
**Rt-DBR2-R**	5' ATAATCAAAACTAGAGGAGTGACCC 3'	qRT-PCR
**Rt-ALDH1-F**	5' CAGTTTCTGACCCAAATCCAGGTTGA 3'	qRT-PCR
**Rt-ALDH1-R**	5' TCGGAGTAGTTGGTCACAT 3'	qRT-PCR
**Rt.YAB5.F**	5'ATTGTTCTTGCGGTGAGTGT 3'	qRT-PCR
**Rt.YAB5.R**	5'AGCCTTCTGAGGATGGTGATG 3'	qRT-PCR
